# Microfluidic Wearable Devices for Sports Applications

**DOI:** 10.3390/mi14091792

**Published:** 2023-09-19

**Authors:** Fangyuan Ju, Yujie Wang, Binfeng Yin, Mengyun Zhao, Yupeng Zhang, Yuanyuan Gong, Changgeng Jiao

**Affiliations:** 1 College of Physical Education, Yangzhou University, Yangzhou 225127, China; 007882@yzu.edu.cn (F.J.); mz120220837@stu.yzu.edu.cn (Y.W.); mz120230818@stu.yzu.edu.cn (M.Z.); 211102236@stu.yzu.edu.cn (Y.Z.); 2 School of Mechanical Engineering, Yangzhou University, Yangzhou 225127, China; binfeng27@hotmail.com; 3 Institute of Physical Education, Shanghai Normal University, Shanghai 200234, China; 1000529125@smail.shnu.edu.cn

**Keywords:** wearable technology, microfluidic technology, physiological signal, in vitro monitoring techniques, wisdom sports

## Abstract

This study aimed to systematically review the application and research progress of flexible microfluidic wearable devices in the field of sports. The research team thoroughly investigated the use of life signal-monitoring technology for flexible wearable devices in the domain of sports. In addition, the classification of applications, the current status, and the developmental trends of similar products and equipment were evaluated. Scholars expect the provision of valuable references and guidance for related research and the development of the sports industry. The use of microfluidic detection for collecting biomarkers can mitigate the impact of sweat on movements that are common in sports and can also address the issue of discomfort after prolonged use. Flexible wearable gadgets are normally utilized to monitor athletic performance, rehabilitation, and training. Nevertheless, the research and development of such devices is limited, mostly catering to professional athletes. Devices for those who are inexperienced in sports and disabled populations are lacking. Conclusions: Upgrading microfluidic chip technology can lead to accurate and safe sports monitoring. Moreover, the development of multi-functional and multi-site devices can provide technical support to athletes during their training and competitions while also fostering technological innovation in the field of sports science.

## 1. Introduction

Recently, there has been increasing interest in developing and applying flexible wearable devices, especially in the sports domain. These devices, characterized by their lightweight, sophisticated design, flexibility, remote operability, portability, and real-time data processing capabilities [1,2,3], were initially designed for health monitoring in the medical sector. However, their application has expanded to the sports arena, where they play a pivotal role in assisting athletes with fatigue monitoring and facilitating tailored training adjustments.

**Figure 1:**
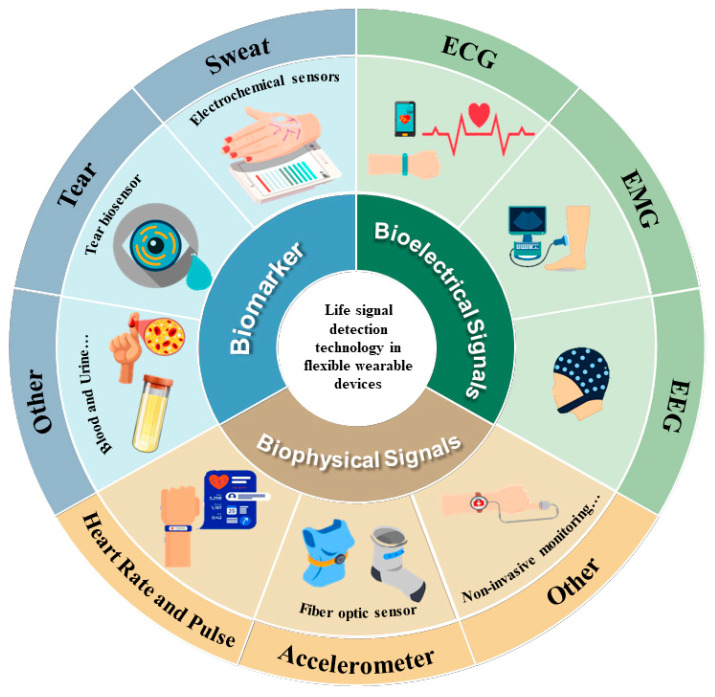
Classification of Vital-Signal-Monitoring via Flexible Wearable Devices in Sports.

Microfluidic chips have emerged as a groundbreaking technology, facilitating the meticulous manipulation and regulation of liquids. This precision is achieved through the intricate design of microchannels, coupled with exact control over liquid flow rates and pressures [4] (refer to Figure 1). Recognizing the potential for enhanced monitoring accuracy, microfluidic chips have been integrated into the design and development of flexible wearable devices. Specifically, these chips empower wearable devices to achieve heightened accuracy in monitoring biochemical, physiological, and bioelectrical signals within a minimized volume [5]. This technological advancement offers a robust foundation for assessing athletes’ training status, monitoring fatigue, and diagnosing and treating sports-related injuries [6,7,8,9]. Furthermore, the compact and pliable nature of microfluidic chips ensures that flexible wearable devices adhere seamlessly to the user’s body. This results in a tailored fit that maintains the user’s range of motion. When paired with soft and lightweight materials, the wearable devices minimize discomfort, even during extended periods of use [6,7,10]. Such ergonomic and functional benefits have solidified the position of flexible wearable devices as indispensable tools in the sports sector.

Recent scholarly endeavors have predominantly centered on the technological evolution and functional enhancements of flexible wearable devices [7,8,10,11,12,13,14]. However, a discernible gap exists in the exploration of the nexus between the functionality of these wearable devices and motor activity.

This study aimed to conduct a comprehensive review of the advancements in flexible wearable technology within the sports domain. This investigation will delve into the intricate interplay between monitoring metrics, functional categorizations, and their implications in sports. Additionally, the study will shed light on the current adoption patterns and prospective trajectories of these devices and equipment in the athletic arena. Through this rigorous examination, scholars aspire to furnish invaluable insights that can steer subsequent studies and catalyze industrial progression.

## 2. Vital-Signal-Monitoring Technology for Flexible Wearable Devices in Sports

Vital-signal detection technology has emerged as a focal point in the realm of wearable sensing technology, underpinning the significance of flexible wearable devices in the sports sector. Leveraging microfluidics, these wearable devices are adept at continuous, non-invasive, real-time monitoring and analysis of a spectrum of biomarkers, biophysical signals, and bioelectrical signals [15,16] (refer to Figure 1). Such capabilities furnish users with instantaneous feedback regarding their physiological status and overall health [9,17]. Consequently, a meticulous exploration of vital-signal detection technology is imperative to further the integration and efficacy of flexible wearable devices within the sports domain. 

### 2.1. Collection of Biomarkers by Microfluidic Chips

**Figure 2:**
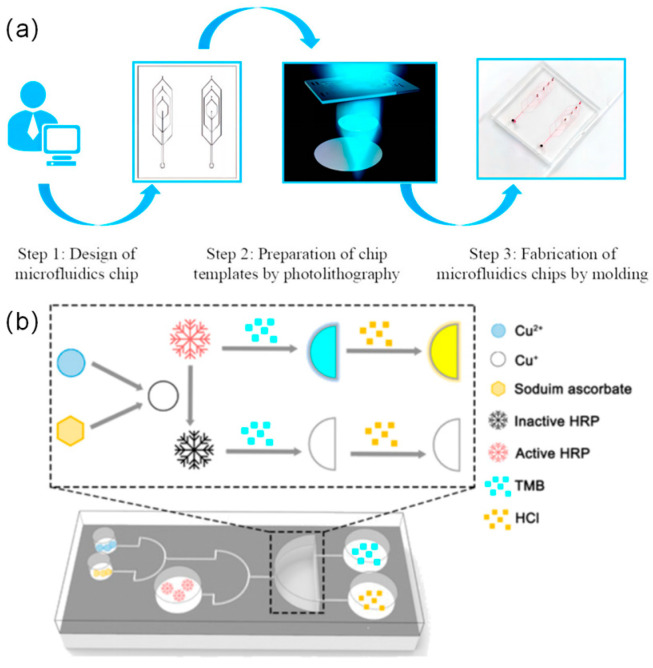
The images of microfluidic chip development and testing process. (**a**) The preparation process of a microfluidic chip [4], copyright (2023), with permission from MDPI. (**b**) The detection mechanism of the fabricated enzyme-method-based microfluidic chip. Reprinted from [18], copyright (2021), with permission from MDPI.

Flexible wearable devices predominantly employ microfluidic detection mechanisms to gather human biomarkers, specifically analyzing athletes’ sweat and tears. The fabrication process and detection mechanism of microfluidic chips for flexible wearable devices are referenced in Figure 2. The fabrication process of microfluidic chips is divided into three steps: Step 1 designs the microfluidic chip template with the help of software; Step 2 uses the fabrication of the microstructure using photolithography and a photoresist (AZ-50XT); and Step 3 joins the polydimethylsiloxane, encapsulation, and drying [4]. Production is complete, and it has been confirmed by testing that the enzyme-based microfluidic chip provides two methods of reading out signals. It can be used to accurately calculate the concentration of Cu^2+^ [18]. This approach facilitates the acquisition of real-time insights into their physiological states [19,20,21]. Such data-driven insights serve as a robust scientific foundation to support athletic training and performance optimization.

#### 2.1.1. Precise Monitoring of Sweat during Exercise

Sweat analysis holds significant appeal for flexible wearable technologies. Given that perspiration is a natural byproduct of exercise, microfluidic chips facilitate the non-invasive and continuous collection of sweat samples over extended periods [22]. The evolution of sweat detection technology in flexible wearable devices has ushered in an advanced phase where sweat is not only harvested via microfluidics but is also scrutinized utilizing both electrochemical and optical sensors. These sweat detection sensors, underpinned by diverse detection methodologies, have been meticulously engineered to enable continuous, in situ analysis of sweat.

Jia (2013) pioneered the development of an electronic tattoo wearable sensor, ingeniously crafted from molecularly imprinted polymers and silver nanowires [23]. It uses electrodes made of treated tetrathiafulvalene (TTF) and multi-walled carbon nanotubes (CNT) coated with biocompatible chitosan. The electrodes are connected to the lactate oxidase (LOx) enzyme, which generates an electrochemical signal when lactate is detected and is used to measure lactate levels. This innovation boasted the distinctive capability of detecting lactic acid with remarkable sensitivity. Building on this foundational technology, Sempionatto (2017) subsequently engineered an electrochemical sensor that discerns lactate, glucose, and potassium ions [24]. This was achieved through the application of screen-printing technology, enabling the differentiation between aerobic and anaerobic exercise states. Notably, this sensor was seamlessly integrated into eyewear, with the device transmitting data to a primary unit via Bluetooth, facilitating the real-time detection of sweat markers. In a subsequent advancement, Zamarayeva (2020) employed flexible printing techniques to create paper-based electrochemical sensors [25]. These sensors incorporated PVC membranes, strategically designed to restrict the diffusion of lactic acid. This innovation ensured that lactic acid detection remained unaffected by sweat flow, thereby enhancing monitoring sensitivity. As electrochemical sensors continue to evolve, flexible wearable devices are achieving unparalleled real-time sensitivity in sweat monitoring. Within the sports domain, this translates to instantaneous reflections of ion shifts, lactic acid variations, and changes in other sweat constituents, paving the way for more precise athletic monitoring.

In a bid to enhance user comfort, there has been a shift towards the development of optical sensors for sweat monitoring. Zhou (2016) pioneered a sweat sensor leveraging gold nanoparticle colloids [26]. This innovative design facilitates rapid differentiation between dehydration and overhydration states by manifesting discernible color changes. This not only augments the visual aspect of monitoring but also marks an initial foray into integrating monitoring technology with textile materials. Building on this foundation, Ardalan (2020) introduced a fluorescence-based wearable patch [27]. Comprising filter paper, cotton thread, and medical tape, this patch is adept at detecting glucose, lactate, pH, and chloride levels [28]. The integration of optical sensors into sweat monitoring devices not only enhances wearability but also bolsters their applicability within the sports domain.

Amidst the progressive advancements in microfluidics and sensor design, flexible wearable technology has adeptly addressed the challenges posed by sweat flow during physical activity. It now boasts the capability to precisely monitor bioindicators present in sweat [29,30,31]. This technological evolution facilitates the identification of exercise intensity thresholds and enables monitoring of the metabolic shift from aerobic to anaerobic states. Such functionalities align well with the requirements of the sports and exercise domain, underscoring the relevance and applicability of these wearable devices.

#### 2.1.2. Safely Monitoring Tears during Exercise

Tears, esteemed as a prime non-invasive diagnostic biofluid, have garnered widespread acknowledgment for their efficacy in monitoring physiological states. This is attributed to their pronounced correlation with the biological constituents found in blood [32,33]. Particularly within the sports arena, the emergence and evolution of flexible wearable tear biosensors have been accentuated. This surge in interest is propelled by technological advancements and the escalating demand for real-time surveillance of athletes’ physiological well-being.

Flexible wearable devices designed for tear monitoring hold distinct advantages in sports applications, primarily due to their placement around the eye. This positioning ensures they are lightweight and minimally intrusive to movement. However, the practical deployment of these tear biosensors in sports settings presents certain challenges. The prototype of the wearable tear biosensor was a flexible strip designed to ascertain glucose levels in tears. This was achieved by coating polydimethylsiloxane (PDMS) immobilized onto poly (MPC-co-DMA) [34]. Yet, its design posed challenges in terms of stable placement on the iris, rendering it less practical for routine wear, let alone rigorous sports activities.

To enhance the wearability of such biosensors, Chu (2011) innovated a contact-lens-like biosensor [35]. This was achieved by immobilizing a glucose oxidase electrode onto a flexible band, thereby facilitating prolonged wear. In the pursuit of optimizing comfort, subsequent research endeavors employed advanced materials, such as graphene–AgNW hybrid materials and titanium dioxide sol–gel membranes [36]. These innovations led to the creation of wearable biosensors resembling contact lenses, offering superior adaptability to the iris. However, given that these contact-lens-like sensors maintain direct ocular contact, there is a pertinent concern regarding potential eye disorders, especially those that might arise from the heat generated by wireless transmission modules. To mitigate the risks associated with direct eye contact, Sempionatto (2019) and colleagues introduced a frame-based wearable tear sensor [37]. This pioneering design, for the first time, showcased the feasibility of detecting extraocular tears. Positioned to gather tears from the eye’s corner, this sensor can concurrently detect glucose, vitamins, and extraocular tears via a bioenzyme–substrate reaction, all while avoiding direct ocular contact.

Through a review of tear and sweat monitoring devices, we compared products for tear detection with those for sweat detection (refer to Table 1) and found that the sensitivity of current products has been improved mainly through improved sensors and that most of the monitoring limitations of the products are due to the effects of changes in biomarker composition. While commendable strides have been made to enhance the comfort, safety, and efficacy of these sensors, especially during physical activity, their vulnerability remains a topic warranting further exploration.

#### 2.1.3. Other Biomarker Monitoring during Exercise

In the field of sports, real-time tracking and assessment of athletes’ physiological status extends beyond just monitoring sweat and tears. Other biomarkers, such as blood, saliva, and urine, have also been harnessed for this purpose.

Blood, replete with a diverse array of bioindicators in high concentrations, stands out as an exemplary diagnostic medium. However, its collection necessitates invasive procedures, posing potential harm to the individual. Consequently, flexible wearable devices tailored for sports often prioritize tear collection to glean biochemical signals. This approach is non-invasive, and studies have corroborated that both blood and tears exhibit analogous compositional shifts, especially when exercise culminates in dehydration [38,39]. Saliva-based sensors have also garnered attention, particularly for disease detection, given their non-invasiveness and minimal risk of contamination or trauma [40]. An example of this is the mouthguard-type uric acid biosensor, which facilitates routine monitoring of uric acid levels in saliva, employing a microcontroller, a constant potentiometer, and Bluetooth low-energy technology [41].

Flexible wearable devices, adept at harvesting biomarkers from non-invasive biofluids like sweat, saliva, and tears, are equipped with biosensing modules that enable continuous and quantitative biosensing. This real-time diagnostic capability holds immense promise in sports, offering precise and instantaneous fatigue monitoring. Such insights can pre-empt fatigue-induced mishaps and bolster athletic prowess.

While blood and saliva biomarker monitoring can indeed reflect physiological alterations during physical exertion, there are inherent challenges. Direct blood monitoring during exercise is impractical due to its invasive nature. Devices monitoring saliva, typically positioned near the mouth, might impede optimal oxygen intake during physical activity. Thus, while the potential of these biomarkers is acknowledged, the development of flexible wearable devices in sports that can effectively harness these biomarkers remains an area ripe for exploration.

### 2.2. Biophysical Signal-Monitoring Technology during Exercise

Recent scholarly investigations have elucidated the utility of biophysical signals in the medical and healthcare sectors for the diagnosis, monitoring, and treatment of a diverse array of ailments [42,43,44]. In the realm of sports, these signals have been predominantly harnessed through the measurement of key indicators such as heart rate, pulse rate, and accelerometer data. Additionally, monitoring mechanisms capturing respiratory patterns, blood oxygen levels, and other vital-signal metrics have also been integrated into sports applications.

#### 2.2.1. Sensitive Monitoring of Heart Rate Information during Exercise

To achieve sensitive and precise cardiovascular health monitoring and mitigate the interference of bodily movements, advancements in flexible devices have incorporated electrical sensing, pressure sensing, and optical sensing. These modalities facilitate the extraction of heart rate and pulse data from regions such as the chest, wrist, neck, and fingers.

Flexible wearable devices have ushered in the era of real-time remote monitoring of ECG, heart rate, and respiration. This is achieved through electrical sensing, which employs methodologies like peak detection and window averaging of ECG signals, facilitated by scalable hybrid electronic systems [45,46]. Compared to electrical sensing, pressure sensors offer the distinct advantage of directly accessing heart rate data. For instance, Rasheed (2017) crafted pressure sensors using a PVDF piezoelectric sandwich structure combined with amorphous silicon bi-gate TFTs, enabling the simultaneous detection of multi-point heart rates [47]. Further, Chen (2022) introduced ultra-responsive/recovery flexible piezoresistive sensors, employing a twisting technique tailored for pulse detection [48]. The inherent benefit of pressure sensors lies in their ability to offer sensitive heart rate monitoring through multipoint measurements, all while ensuring reduced resistance and enhanced comfort. An even more user-friendly monitoring approach leverages photoelectric plethysmography (PPG) to discern volume shifts in blood flow through the skin, thereby extracting heart rate data. Scardulla (2020) delved into the contact pressure dynamics between the PPG sensor and the skin [49]. Building on this foundation, Wang (2021) conceptualized a PI interface sensor, amalgamating a platinum film thermistor with a reflective PPG sensor, designed to gauge the contact pressure between the sensor and the skin [50]. The refinement of PPG technology has enabled sensors to adopt a more streamlined architecture, ensuring that flexible wearable devices retain their flexibility, comfort, softness, and mechanical compatibility, all of which are pivotal for sustained monitoring.

In summation, the trajectory of heart rate and pulse monitoring technology in flexible wearable devices has transitioned from rudimentary electrical sensing to advanced pressure and optical sensing modalities. This evolution minimizes the perturbations from physical activity on monitoring data, thereby enhancing both the sensitivity and precision of the readings.

#### 2.2.2. Intelligent Monitoring of Movement Trajectories during Exercise

The accelerometer, a pivotal tool in sports applications, spans domains from training and competition to health monitoring and entertainment. By precisely gauging acceleration and trajectory, it provides both athletes and general users with an enriched, personalized sports experience.

Incorporated into flexible wearable devices, accelerometers are underpinned by a myriad of mechanisms. Wearable sensors, leveraging principles such as piezoelectricity, triboelectricity, piezoresistivity, and capacitance, have been conceptualized to monitor bodily movements, including finger flexion. These sensors stand out for their commendable attributes, encompassing high sensitivity, robustness, and flexibility. Building on this foundation, Wang (2021) devised stretchable, self-adhesive strain sensors tailored for monitoring movements across the ankle, wrist, and neck [51]. These sensors, featuring a dry adhesive layer crafted from waterborne polyurethane, adhere seamlessly to the skin, offering heightened sensitivity and markedly diminishing motion artifacts. In a subsequent innovation, Bi (2020) employed a conductive fabric coated with rGO/carbonate ink/PVA, to monitor limb movements [52]. This fabric adheres comfortably to the skin and, when integrated with algorithms, can be harnessed for posture correction in elite athletes. Advancing this technology, Zhao (2021) introduced a highly stretchable, transparent, wearable ionic skin system [53]. Affixed to the hand, this system can discern various gestures and relay them to smartphones via wireless communication, offering a novel avenue for sign language recognition, thereby aiding the deaf and speech-impaired in communication. Furthermore, some researchers have tailored accelerometers specifically for foot movement. For instance, Mao (2021) crafted a smart sock, integrating a TENG, signal pre-processing circuits, and a microcontroller with a wireless transmitter [54]. This sock is adept at monitoring plantar pressure and, through neural network algorithms, can analyze and monitor gait and stability.

Wearable sensor technology has transitioned from rudimentary mechanisms to a stage characterized by multifunctionality and heightened sensitivity. These advancements cater not only to basic movement monitoring but also to specialized sports training and motion tracking. As artificial intelligence continues to evolve, accelerometers in flexible wearable devices, complemented by algorithmic analysis, are poised to offer intelligent monitoring, transcending the challenges posed by motion artifacts and perspiration.

#### 2.2.3. Continuous Monitoring of Respiration and Blood Oxygen during Exercise

In the realm of sports, commonly monitored biophysical signals extend beyond heart rate to encompass respiration, body temperature, and blood pressure. Given the emphasis on continuous monitoring, the evolution of flexible wearable technology in this domain seeks to address challenges related to device–skin fit and ensure uninterrupted, precise data capture.

Researchers have pioneered continuous respiratory monitoring by amalgamating pressure and stretch sensors, allowing the device to snugly adhere to the skin, thereby tracking chest movements during inhalation and exhalation [6,55,56]. Such advancements hold immense potential for extended monitoring and optimizing athlete training regimens. Concurrently, the integration of optical sensors into flexible wearable devices, bolstered by the advent of pliable optical fibers and organic optoelectronics, has rendered these devices more lightweight and conducive to extended wear. Illustratively, Wang (2018) introduced a stretchable optical sensing system capable of concurrently monitoring heart rate, blood oxygen saturation, and sweat pH, even on the body’s contoured surfaces [57]. Such innovations not only enhance health surveillance but also herald transformative shifts in medical and sports health management. Furthermore, body temperature sensors have been seamlessly incorporated into flexible wearable devices. For instance, Jin (2020) devised a cost-effective flexible sensor that tracks skin temperature fluctuations in real time and extracts pulse waves, enabling the identification of physiological states such as movement, excitement, and fatigue [58]. Chen (2020) highlighted that conventional temperature measurement techniques, which necessitate pressing the device against the body, might inadvertently alter local body temperature, thereby compromising accuracy [59]. In contrast, skin-mimicking flexible wearable devices can unobtrusively measure skin temperature with heightened precision, offering superior solutions for sustained temperature monitoring.

Beyond respiratory and temperature tracking, flexible wearable devices are also demonstrating potential in non-invasive blood pressure monitoring. Such technology gauges blood pressure by assessing pulse wave velocity and other vascular metrics. Its portability and continuous monitoring capabilities present novel avenues for cardiovascular health oversight [60,61,62]. While research in this domain is ongoing, the prospective contributions of flexible wearable devices to non-invasive blood pressure monitoring are eagerly awaited.

In refining the monitoring techniques of respiration, body temperature, and blood pressure within flexible wearable devices for sports, the emphasis predominantly lies on ensuring continuous monitoring and optimizing device–skin compatibility. This dual focus aims to enhance both the accuracy of prolonged monitoring and the comfort of device wearability. However, it is noteworthy that, during physical activity, signals from body temperature and blood pressure offer a limited snapshot of physiological status, which might explain the relative scarcity of such flexible wearable devices.

### 2.3. Bioelectrical-Signal-Monitoring Technology during Exercise

In the field of sports, flexible wearable devices predominantly focus on monitoring three core bioelectrical signals: ECG (electrocardiography), EMG (electromyography), and EEG (electroencephalography). These signals collectively offer a comprehensive representation of various physiological indicators. Compared to biological and biochemical signals, the acquisition of bioelectrical signals is more straightforward. This simplicity, coupled with their comprehensive nature, positions bioelectrical signals as having immense potential and broader applicability in the sports sector.

#### 2.3.1. Lightweight Monitoring of Heart Function during Exercise

In the medical realm, various ECG instruments have been devised to record the heart’s electrical activity, predominantly utilizing wet electrodes [12,63]. While these medical-grade ECG monitors provide precise data, their design is tailored for short-term stationary assessments. Their intricate operational procedures and substantial size render them ill-suited for dynamic settings like sports.

ECG monitoring is pivotal in evaluating cardiovascular health and exercise endurance in athletes. Consequently, there’s a pressing need for innovations in flexible wearable technologies tailored for ECG assessments. Addressing this, Kim (2018) introduced a novel flexible wearable device for ECG monitoring [64]. Leveraging thin-film electronic layers combined with superelastic elastomers, this device seamlessly adheres to the skin, facilitating prolonged wear and uninterrupted data acquisition during physical activity. Its hallmark lies in its adaptability and snug fit, ensuring efficient, accurate data collection with minimal inconvenience to the wearer. Building on this foundation, Luo’s WOC-SVM monitoring technology harnessed ECG signals to gauge fatigue levels and detect anomalies during physical exertion [65]. Through specialized algorithms and data processing techniques, this system offers real-time fatigue assessments, delivering timely alerts for fatigue onset and potential irregularities. Aimed at refining this monitoring paradigm, both Ren (2017) and Asadi (2020) concentrated on detecting subtle heart rate variations [66,67]. Their continuous technological enhancements sought to mitigate the impacts of skin resistance and perspiration during exercise, ensuring accurate, real-time monitoring.

Flexible wearable ECG monitors tailored for sports applications are inherently more compact and user-friendly than their medical counterparts. Through relentless technological advancements, these devices are progressively minimizing external interference, making them increasingly apt for the dynamic environment of sports.

#### 2.3.2. Integrated Monitoring of Muscle Function during Exercise

Surface electromyography (sEMG) monitoring traditionally necessitates a consistent electrical connection to the skin. This connection is often vulnerable to environmental factors and skin impedance variations. Historically, achieving this connection has involved the use of microneedles inserted into the muscle or the application of wet electrodes [11]. However, the evolution of EMG sensors tailored for sports has streamlined the monitoring process. These modern sensors not only facilitate the capture of muscle function metrics but also track movement trajectories, offering invaluable assistance for sports, rehabilitation, and healthcare domains.

**Figure 3:**
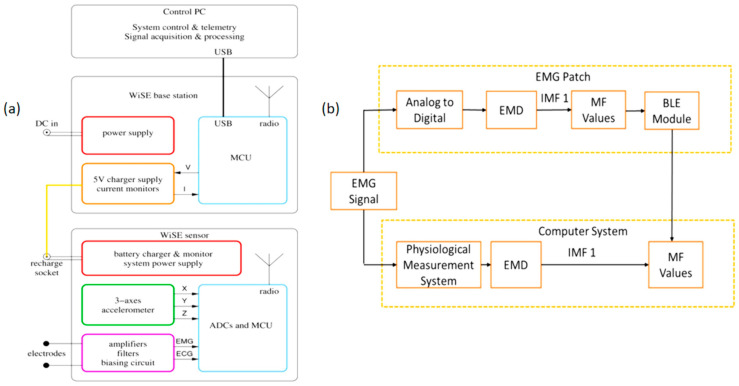
The images of different EMG research and development ideas. (**a**) WiSE system block diagram. reprinted from [68], copyright (2018), with permission from SPRINGERLINK. (**b**) The framework of comparison between the real-time EMG patch and the offline computer system. Reprinted from [69], copyright (2019), with permission from MDPI.

The contemporary focus in the realm of sports-based EMG monitoring emphasizes the holistic development and deployment of EMG technologies across diverse application scenarios (Figure 3). For instance, Biagetti (2018) introduced a cost-effective wearable wireless system explicitly crafted to capture sEMG and accelerometer data [68]. This system was envisioned to monitor human activity across sports, fitness, and healthcare domains. In a parallel endeavor, Liu’s team (2019) pioneered an sEMG patch designed for placement on the calf’s gastrocnemius muscle [69]. This innovation aimed to detect real-time muscle fatigue during physical activity, offering a proactive approach to preventing injuries stemming from muscle exhaustion. Further emphasizing the significance of sEMG in rehabilitation and health monitoring, Al-Ayyad et al. (2023) conducted a comprehensive review of wearable sEMG devices [70]. Their analysis, which underscored the technical specifications and wearability of these devices, highlighted their current applications in rehabilitation exercises and physiological monitoring.

Collectively, these advancements underscore the pivotal role of EMG monitoring in the sports sector. As a tool, it offers a nuanced assessment of muscle functionality, facilitates human activity tracking, and aids in rehabilitation exercises. The multifaceted capabilities of flexible wearable EMG monitors herald a new era of personalized, remote health monitoring, enriching our comprehension and management of muscle-centric conditions and activities.

#### 2.3.3. Effective Monitoring of Neural Function during Exercise

Electroencephalography (EEG) monitoring in the medical domain traditionally employs wet electrodes. However, this method presents challenges, including interference from hair, inter-electrode disturbances, and patient discomfort [71]. As flexible wearable technology advances, research in the sports sector is increasingly exploring innovative EEG monitoring techniques. These aim to mitigate the effects of hair and movement, ensuring efficient monitoring.

Numerous studies have delved into alternative methods, such as needle electrodes and capacitive sensors, to enhance EEG detection. Liao (2011) pioneered a spring-loaded contact probe structure, utilizing needle-like electrodes that penetrate the hair to establish direct skin contact [72]. This design effectively circumvents the interference posed by hair. Building on this, Ren (2020) crafted electrodes with lower-impedance columnar microneedles, integrating them with headphones [73]. This innovation facilitated the capture of high-quality, stable EEG signals over extended periods. Beyond needle electrodes, the realm of flexible wearable devices has also embraced non-contact EEG detection via capacitive sensors. This approach minimizes direct sensor–hair contact, further reducing hair-related interference. Chi et al. (2009) were pioneers in this space, developing a non-contact capacitive sensor capable of effectively capturing EEG signals and wirelessly transmitting them to smart devices for real-time monitoring and visualization, enhancing user comfort [74]. Subsequent research by the same team led to the integration of these sensors into the fabric, culminating in the creation of wireless, non-contact EEG-monitoring headbands [75]. Carneiro (2020) further refined this technology, introducing a second layer of contact electrodes to mitigate noise from extended wires and electromagnetic interference (EMI) [76]. They combined this with the Internet of Things (IoT) technology, resulting in an EEG headband equipped with printed silver-based conductive fabric electrodes. This evolution in EEG detection has also given rise to diverse monitoring devices, including smart earplugs [77] and embedded eyepieces [78].

In their current iteration, flexible EEG monitoring devices are primarily suited for the daily monitoring and motor rehabilitation of individuals with neurological disorders. By assessing the neural activity of the brain’s neocortex, these devices can infer motor intentions, thereby aiding in rehabilitative training.

## 3. Classification of Flexible Wearable Devices for Sports Applications

**Figure 4:**
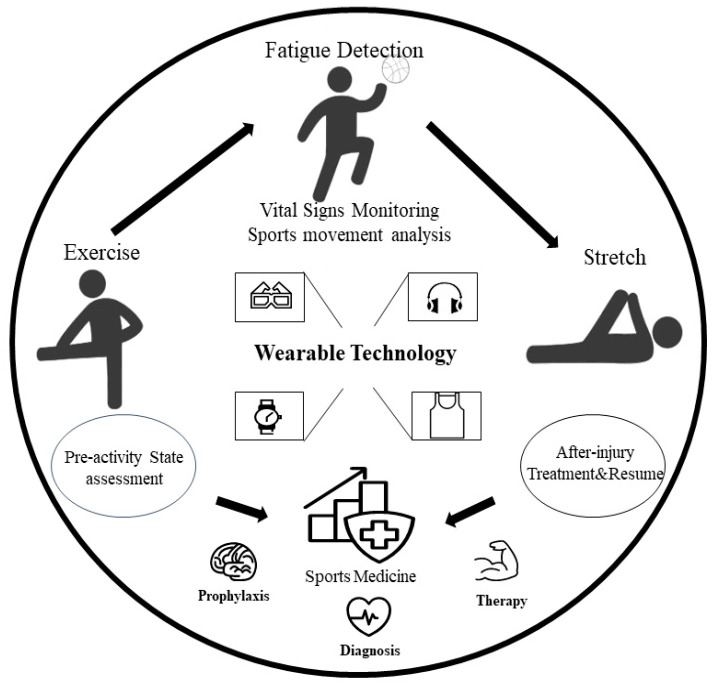
Classification of applications of flexible wearable devices in the field of sports.

As illustrated in Figure 4, flexible wearable devices, characterized by their lightweight design, adaptability, and ergonomic fit, offer transformative experiences and augmented capabilities to both amateur sports enthusiasts and professional athletes across domains such as sports training, sports medicine, and active participation.

### 3.1. Functional Classification in the Field of Athletic Training

In the realm of athletic training, flexible wearable devices have garnered significant attention and adoption. These devices are pivotal in augmenting athletic prowess, mitigating injury risks, and fostering sustained well-being. They facilitate foundational health evaluations, instantaneous kinematic analyses [14], and tailored recuperative strategies.

#### 3.1.1. Pre-Exercise: Condition Assessment

In the domain of sports and physical training, flexible wearable devices have emerged as indispensable tools for gauging the physical preparedness of participants. Moatamed (2017) introduced a monitoring framework utilizing wearable jump sensors to measure heart rate variability (HRV) [79]. By examining the correlation between HRV and physiological markers, this system offers a nuanced assessment of an individual’s physical state prior to exercise. Zadeh (2021) integrated data analytics with wearable technology, employing the Zephyr BioHarness device to capture quantifiable metrics related to body composition in active individuals [80]. Their analysis discerned the interplay between these metrics and exercise load, aiming to mitigate injury risks. Such technological integrations pave the way for the formulation of evidence-based exercise regimens, minimizing the potential for load-induced injuries.

Piłka (2023) underscored the potential of flexible wearable devices in crafting individualized training protocols, with the dual objective of averting sports-related injuries and optimizing training efficacy [81]. Their methodology encompassed a decision-making framework reliant on GPS-enabled wearable sensors to collate data during training or competitive events. This model not only gauges potential injury-inducing loads in subsequent training sessions but also calibrates the training regimen by recommending optimal load allocations, ensuring athletes’ safety and performance enhancement.

Distinct from the aforementioned research, Ning (2023) ventured into the realm of physical education, proposing an Internet of Things (IoT)-centric monitoring and training system [82]. Aimed at curtailing sports injuries among students, Changfeng Ning’s design harnesses the IoT to amass student-centric data during physical activities. This system meticulously processes and categorizes the data, evaluates students’ fitness profiles, pinpoints potential issues, and proffers remedial measures, thereby fortifying injury prevention in sports pedagogy.

In summation, flexible wearable devices stand at the forefront of pre-exercise physical evaluations. Numerous studies corroborate that the judicious amalgamation of objective data from wearable devices with subjective athlete feedback can enrich our comprehension of their physiological state. Such insights empower athletes to adhere to scientifically validated training paradigms, substantially diminishing injury risks.

#### 3.1.2. In Exercise: Fatigue and Movement Monitoring

In the realm of sports science, real-time assessment of athlete fatigue has been revolutionized by the advent of flexible wearable devices. These devices meticulously monitor pivotal metrics such as lactate threshold and heart rate variability (HRV), offering insights into an athlete’s physiological state. Furthermore, the real-time analysis of athletes’ kinematics and posture is now feasible through advanced motion capture and sports biomechanics modeling techniques [83]. Such analyses facilitate the fine-tuning of training intensities, acting as a bulwark against overtraining.

The efficacy of flexible wearable devices in the timely detection of exercise-induced fatigue is well documented. By analyzing a spectrum of physiological metrics, these devices provide a nuanced understanding of an athlete’s physical condition, enabling tailored adjustments to exercise regimens. This proactive approach not only mitigates the risk of overtraining but also significantly reduces injury susceptibility [84,85]. As a notable advancement, Sultan and Abbosh (2022) introduced a wearable dual-polarized electromagnetic system tailored for knee imaging [86]. This innovative system holds the promise of continuous monitoring of knee health, allowing for early detection and intervention of potential injuries, thereby minimizing the risk of injury aggravation. Lu (2021) innovatively incorporated nanogenerator sensors into the equipment of skiers [87]. These sensors produced distinct motion-induced voltage signals corresponding to varied movement states, enabling a granular analysis of parameters like skating angle and technique. Such insights have the potential to significantly enhance training methodologies and overall athletic performance [87,88].

However, it is imperative to acknowledge the inherent variability in physical responses to fatigue, which is contingent on individual idiosyncrasies. Consequently, model construction and subsequent data interpretation necessitate a personalized approach [88]. External factors, including sweat production, variations in device placement, and equipment sensitivity, can introduce discrepancies in the monitoring process. The integration of monitoring technology into sports equipment has further expanded the horizons of biomechanical analysis.

While flexible wearable devices have made significant strides in monitoring fatigue and biomechanics in sports, there remains a challenge in achieving personalized monitoring criteria due to the current limitations in device intelligence. This underscores the need for further advancements in the domain, particularly focusing on individualized sports fatigue monitoring.

#### 3.1.3. After Exercise: Fatigue Recovery

Emerging at the forefront of technological innovation, flexible wearable devices present a paradigm shift in the real-time monitoring of human biomechanical and physiological parameters. These devices adeptly capture a spectrum of dynamic metrics encompassing speed, endurance, coordination, agility, joint range of motion, heart rate, and respiratory rate. Their versatility and precision render them invaluable tools for post-exercise rehabilitation monitoring.

The real-time capabilities of flexible wearable devices extend beyond mere athletic performance metrics. They offer profound insights into joint health by meticulously tracking the range of motion, and they provide a nuanced understanding of cardiorespiratory function through the continuous monitoring of heart and respiratory rates [15,84,89]. Liu (2023) unveiled a state-of-the-art wearable graphene heating device [90]. Harnessing the therapeutic potential of far-infrared radiation, this device is specifically designed to expedite the recovery of bicep fatigue. When juxtaposed with conventional recovery modalities such as massage, hydrotherapy, cryotherapy, and electrical stimulation, this innovative wearable device stands out for its energy efficiency, user-centric design, and wearability.

While the medical domain offers sophisticated and precise fatigue detection post-exercise, the associated equipment often comes with a hefty price tag and is predominantly reserved for clinical applications. The operation of such equipment typically necessitates the expertise of healthcare professionals, thereby constraining its broader applicability. In contrast, while flexible wearable devices are still in their nascent stages of development for rehabilitation purposes, their potential is undeniable. They hold the promise of democratizing post-exercise recovery monitoring, making it more accessible and user-friendly for the masses.

### 3.2. Functional Classification in the Field of Sports Medicine

Flexible wearable devices, transcending their conventional applications, are increasingly pivotal in the realm of sports medicine. Beyond merely monitoring fatigue during athletic training, these devices are instrumental in both the diagnostic and therapeutic dimensions of sports injuries. By leveraging the capabilities of these wearable devices, practitioners can achieve prompt and precise diagnosis of injury manifestations, thereby facilitating targeted and efficacious treatment interventions.

#### 3.2.1. Sports Injury Prevention

The prevention of sports injuries has become a focal point of recent research, with an emphasis on the utilization of flexible wearable devices for continuous motion detection and early warning systems. Numerous studies have underscored the potential of wearable devices for consistently monitoring human kinematics [91,92]. A predominant instrument in this realm is the strain sensor, which operates based on the detection of alterations in substrate resistance [93]. Such continuous surveillance and periodic analysis can discern anomalies in an individual’s biomechanics during physical activities, thereby mitigating the risk of sports-related injuries.

In the contemporary landscape of wearable technology, pressure sensors have found prominence, especially in devices tailored for larger joints such as the elbow, knee, and ankle. Di Tocco (2021) ingeniously embedded metal in elastomers to engineer a pressure sensor [94]. This sensor, strategically positioned on the hip, knee, and ankle joints, is adept at monitoring their flexion angles. Such a design offers the dual advantage of precision in tracking joint flexion angles and providing insights into the user’s physiological state. Nonetheless, a potential drawback is the prolonged exposure to metal electrodes, which might instigate dermatological concerns.

To address this challenge, recent advancements have seen the emergence of flexible strain sensors. M. He (2021) seamlessly incorporated sensing components into textiles, optimizing the monitoring of knee joint movements [95]. Their versatility lies in their customizability, allowing for varied sizes and configurations to cater to the comfort preferences of individual users. Shu’s team developed devices that, equipped with a Triboelectric Nanogenerator (TENG) yarn, capture body movements by affixing highly adaptable and extensible TENG fabrics to specific body regions, such as the arms and knees [96]. This device’s hallmark is its superior skin compatibility and durability, making it an ideal choice for athletes’ prolonged usage.

In summation, through the adept integration of pressure sensors, flexible wearable devices have the prowess to monitor biomechanical force variations across the human body. This capability is instrumental in enhancing activity monitoring and preemptively averting injuries. However, a salient challenge remains: the intricate technology underpinning these devices results in elevated production costs, posing a barrier to large-scale manufacturing.

#### 3.2.2. Sports Injury Diagnosis

The evolution of wearable sweat biosensors has been significantly influenced by the advancements in flexible wearable devices, particularly in the context of sports injury diagnostics. Sweat, rich in metabolites and electrolytes, has been identified as a potential reservoir of biomarkers for various diseases, offering innovative avenues for early injury detection.

Athletes and individuals involved in rigorous physical training can leverage these biosensors to obtain real-time insights into their physiological state. The biochemical composition of sweat undergoes dynamic changes during physical exertion, especially at elevated temperatures or during intense activities [97]. This results in the depletion of water and essential electrolytes. By continuously monitoring these changes, athletes can promptly discern alterations in their physiological status. This timely detection empowers them to implement immediate interventions, mitigating further injuries or potential health complications [98].

Moreover, these biosensors offer a comprehensive evaluation of the physiological impact of training regimens. Post-exercise, the analysis of variations in sweat metabolites and electrolytes provides athletes with a nuanced understanding of their fatigue levels and recuperation rates. Such insights are instrumental in tailoring training schedules and recovery protocols to align with individual needs. Furthermore, the continuous surveillance of sweat can preemptively identify exercise-induced health anomalies, such as muscular injuries or persistent inflammation. The early detection of specific biomarkers in sweat can herald potential health threats, enabling timely interventions to preserve the health and longevity of athletes’ careers [99,100].

Wearable sweat biosensors represent a paradigm shift in the realm of sports injury diagnostics. They furnish athletes with a continuous stream of physiological data, facilitating a deeper comprehension of their physical state. This real-time feedback mechanism allows for the calibration of training and rehabilitation regimens, ensuring optimal performance and health. Furthermore, their potential for early disease detection underscores their pivotal role in safeguarding the health and careers of athletes.

#### 3.2.3. Sports Injury Treatment

The realm of bioelectrical-signal-based therapeutic tools has witnessed remarkable advancements, especially in the context of flexible wearable devices tailored for injury management [101].

Electrostimulation, a therapeutic modality, has been extensively employed to address a spectrum of conditions ranging from severe movement disorders and pain to depression and skin regeneration. Within this domain, wearable and implantable electrotherapy devices (WIEs) have emerged as pivotal instruments. These devices are engineered to deliver electrical impulses to specific organs and tissues for therapeutic outcomes. The design imperatives for WIEs encompass miniaturization, adaptability, biocompatibility, and, in certain instances, biodegradability, ensuring both patient comfort and safety [102]. Contemporary research in this field is pivoting towards the development of wearable and implantable power sources, state-of-the-art adaptive electrodes, and efficacious electrical stimulation methodologies. These innovations aim to modulate the aberrant activities within neural networks, thereby eliciting therapeutic benefits [103].

The evolution of electrical stimulation technologies necessitates addressing the intricate engineering challenges inherent to wireless implantable electrodes (WIEs) that facilitate therapeutic electrical stimulation (ES). The engineering paradigms guiding the development and clinical translation of WIEs encompass attributes such as functionality, mirroring capabilities, adaptability, biometric compatibility, and biodegradability [104]. It is imperative to elucidate technical abbreviations upon their inaugural mention for clarity. Presently, the innovation trajectory for WIEs is gravitating towards wearable and implantable power sources, cutting-edge adjustable electrodes, and precision-targeted electrical stimulation. A burgeoning area of research within this domain is the exploration of nano-generators as potential power sources for wearable and implantable devices. These nano-generators promise a consistent and sustainable energy reservoir for WIEs [105]. Concurrently, electrode research is gaining momentum, with the objective of optimizing its adherence to target tissues and ensuring efficient electrical signal transmission, thereby amplifying therapeutic efficacy.

The integration of WIEs within the medical landscape augments the spectrum of patient care, offering bespoke and efficacious treatment modalities. Specifically, in sports medicine, wearable technology holds the potential to significantly ameliorate movement disorders and expedite the convalescence from sports-related injuries. Nonetheless, this technology is not devoid of challenges, predominantly in terms of technical intricacies and safety concerns. Continuous iterations and enhancements are imperative to bolster both the safety and efficacy of these devices.

### 3.3. Flexible Wearable Devices Provide Assistance to Diverse Populations in Terms of Sports Participation

Presently, flexible wearable devices have become integral to the training and rehabilitation protocols of numerous athletes. Concurrently, there is an expanding frontier of research and development focused on creating devices tailored to diverse demographic groups, facilitating their active participation in sports. This study collates and examines the application of flexible wearable devices across distinct populations, including students, the elderly, and individuals with special needs, with an emphasis on analyzing their specific functionalities.

#### 3.3.1. Helping Young People Prevent Sports Injuries in School Physical Education

In the contemporary educational landscape, technological advancements have progressively permeated physical education, enhancing pedagogical methodologies. Consequently, there is a burgeoning development of wearable technology tailored specifically for the younger demographic.

In a seminal study by Cui, Du, and Wu (2023), the potential of wearable devices to capture motion data was explored [106]. This data, when transmitted through the Internet of Things (IoT) and subsequently processed using time-series analysis, machine learning algorithms, and artificial neural networks, offers profound insights. The study delves into the intricate relationship between motion data and the facets of physical recovery and injury prevention. The integration of such technology can provide empirical guidance to physical education curricula, bolstering injury prevention measures. This, in turn, elevates the quality of physical education, fostering enhanced physical recuperation capabilities among young people.

Additionally, wrist-worn wearable devices have emerged as potent tools for adolescents, enabling self-regulation and monitoring of their physical activity, thereby promoting increased activity levels (Hao et al., 2021 [107]). Guo (2022) underscored the potential of microfluidic devices for non-invasive and continuous assessment of the physical health of adolescents [108]. This approach facilitates the early identification of potential health anomalies and monitors the efficacy of fitness interventions. Such a model can heighten adolescents’ cognizance of their physical well-being, catalyzing the adoption of salubrious lifestyle habits.

In the context of school sports, flexible wearable devices have emerged as pivotal instruments in sports injury prophylaxis. The incorporation of microfluidic devices, offering real-time surveillance and feedback, empowers adolescents to optimize their health and mitigate injury risks. Indisputably, the integration of flexible wearable devices furnishes robust technological scaffolding, championing the holistic well-being and healthy maturation of the younger generation.

#### 3.3.2. Helping Older Adults Monitor Their Health Status during Daily Exercise

In the contemporary healthcare landscape, flexible wearable devices have emerged as indispensable instruments in the health management of the elderly population. Particularly for individuals grappling with chronic ailments, these devices, underpinned by microfluidic chips, facilitate the real-time monitoring of pivotal physiological parameters such as body temperature and blood pressure. Such continuous surveillance expedites the early detection and intervention of potential health anomalies, thereby enhancing the efficacy of treatments and mitigating complications [109,110,111].

A notable innovation in this domain is the wearable skin-like sensor developed by Huang (2022) [112]. This sensor, rooted in microfluidic design, is designed for seamless integration with clothing or direct application to the skin [112]. It facilitates real-time monitoring of physiological signals, enabling early identification and management of health concerns. Such an intuitive health monitoring modality is especially beneficial for the elderly cohort, given their potential susceptibility to chronic conditions and the requisite for consistent health surveillance.

In the realm of home-based care, flexible wearable devices stand out as pivotal tools that ensure the safety and well-being of the elderly. These devices, when interfaced with mobile platforms, empower healthcare professionals to remotely monitor the health metrics of the elderly, promptly identifying emergencies such as potential falls [113,114,115]. The integration of microfluidic chips within these wearable sensors not only augments the comfort and safety of the elderly but also offers invaluable peace of mind to their caregivers and families.

In general, the incorporation of microfluidic chips within wearable devices heralds a transformative shift in elderly care. These devices provide comprehensive and efficient health monitoring, elevating the quality of life for senior citizens. By fostering heightened health awareness and self-management capabilities, they provide a robust framework for safeguarding the health and well-being of the elderly population.

#### 3.3.3. Providing Conditions for Special Populations to Participate in Sports

Flexible wearable sensors epitomize the zenith of technological advancements, significantly enhancing the quality of life for individuals with disabilities. These devices herald a transformative era, enabling disabled athletes to engage in sports activities in a non-invasive and portable fashion.

A notable application of flexible wearable sensors is their integration into a rehabilitation system equipped with a virtual reality (VR) gaming interface. This system is tailored for stroke patients with upper and/or lower extremity impairments, facilitating autonomous home-based rehabilitation [116]. This innovative approach not only diminishes the logistical challenges associated with rehabilitation but also empowers therapists to remotely monitor patients’ health trajectories in real time. The synergistic amalgamation of VR gaming with wearable technology infuses an element of enjoyment into the rehabilitation journey, transforming it from a potentially monotonous regimen into an engaging experience. Furthermore, advancements in smart cities and smart home infrastructures are crafting environments that prioritize accessibility, enabling individuals with disabilities to lead more autonomous lives [117].

Zeagler (2018) introduced a smart assistive wearable device specifically designed for the deaf–blind community, exemplifying the pinnacle of human-centric design [118]. These devices, leveraging tactile and thermal communication through soft interfaces and textiles, are revolutionizing the way the deaf–blind community interacts with the world. Concurrently, wearable sensors, including smart gloves and advanced prosthetics, are empowering individuals with disabilities to operate motorized devices, facilitating the transmission of tactile sensations and even pain. Such innovations not only enable them to navigate the world more freely but also enrich their experiential spectrum [119]. The deployment of flexible wearable devices for individuals with disabilities transcends mere technological triumphs; it embodies a profound manifestation of compassionate care.

In conclusion, the invaluable contributions of flexible wearable devices in terms of enhancing the sports participation experience for individuals with disabilities are undeniable. These devices amplify opportunities for engagement in sports, elevate the quality of their athletic experiences, and empower them with real-time insights into their physiological states through cutting-edge microfluidic design.

## 4. Flexible Wearable Device Product Categories in Sports

This research systematically collates flexible wearable devices specifically engineered for sports applications. The devices have been methodically categorized based on their respective wearable locations, with succinct descriptions provided for their functionalities. Comprehensive details pertaining to these devices are delineated in Table 2.

### 4.1. Flexible Wearable Devices Applied to the Head

**Figure 5:**
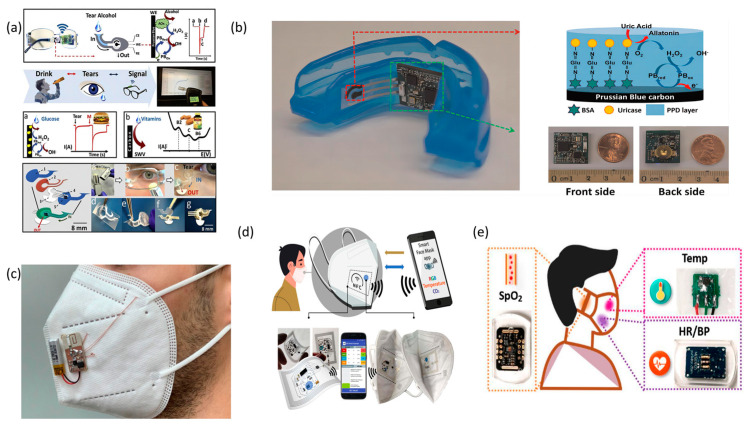
The images of flexible wearable products worn on the head. (**a**) Eyeglasses-based fluidic device. Reprinted from [37], copyright (2019), with permission from Elsevier. (**b**) Photograph of the mouthguard biosensor integrated with the wireless amperometric circuit board and its component. Reprinted from [120], copyright (2015), with permission from Elsevier. (**c**) Smart mask prototype. Reprinted from [121], copyright (2021), with permission from IEEE Xplore. (**d**) Photograph of the NFC-based smart facemask for wireless CO_2_ real-time determination. Reprinted from [122], copyright (2022), with permission from Nature Communications. (**e**) Schematic of the LOM. Reprinted from [123], copyright (2020), with permission from ACS Publication.

The domain of flexible wearable devices is witnessing a dynamic expansion in its application spectrum, with head-worn devices emerging as a novel frontier. Devices tailored for head application prioritize lightweight construction and ergonomic design, rendering them especially apt for sports-related endeavors (Figure 5).

Sempionatto (2019) pioneered a sensing platform integrated into eyeglasses [37]. This innovative design employs nasal pad electrodes in direct contact with the skin, facilitating the monitoring of sweat metabolites and electrolytes. A fluidic device, strategically positioned on the glasses’ nose pad, facilitates the direct collection of stimulated tears. Analytical processes are executed via an alcohol oxidase (AOX)-based electrochemical detector. The ingenuity of Sempionatto et al.’s design lies in its transformation of conventional eyeglasses into a potent biomonitoring apparatus. In a parallel development, Kim (2015) introduced a groundbreaking wearable mouthguard biosensor [120]. This device is adept at real-time monitoring of uric acid levels in saliva, amalgamating a wireless amperometric circuit with an on-chip Bluetooth low-energy communication system. Its design emphasizes miniaturization and energy-efficient operation, underscoring its potential in sports health surveillance.

Furthermore, the advent of smart face masks equipped with heat flux sensors marks a significant stride in wearable technology. Lazaro (2021) and colleagues conceptualized a smart mask that is capable of non-invasively monitoring body temperature and respiratory rate [121]. The integrated system can archive data within the microcontroller’s memory, facilitating subsequent retrieval and analysis. Complementing this, the wireless CO_2_ monitoring smart face mask proposed by Escobedo (2022) and the Lab-on-Mask conceptualized by Pan (2020) for respiratory pattern surveillance further enrich the smart mask technological portfolio [122,123].

The integration of flexible wearable devices for head applications heralds a transformative shift in daily life conveniences. Whether manifested as eyeglass-integrated sensing platforms, wearable mouthguard biosensors, or an array of smart masks, these innovations epitomize the confluence of technology and health, opening new avenues for sports and health monitoring.

### 4.2. Flexible Wearable Devices Applied to the Upper Body

**Figure 6:**
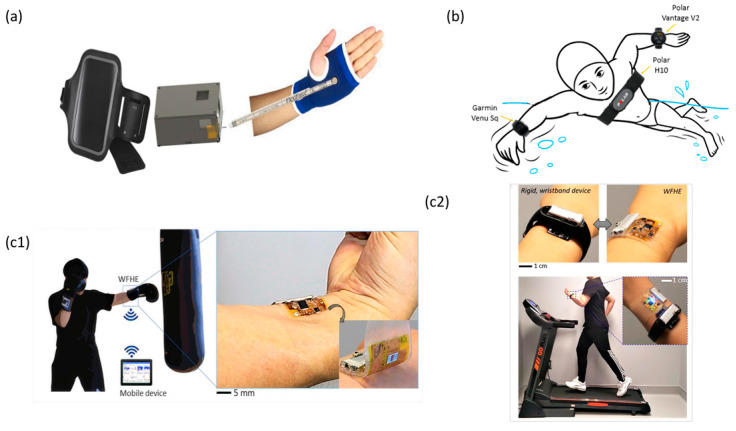
The images of flexible wearable products worn on the upper body. (**a**) A schematization of the device and its components as arranged on the forearm of an athlete during performance. Reprinted from [124], copyright (2019), with permission from RUA. (**b**) Test chart of the cardiac belt (used as reference instrument) and two smartwatches. Reprinted from [125], copyright (2022), with permission from MDPI. (**c1**) Test chart of Wearable Flexible Hybrid Electronics (WFHE) for health and performance monitoring. (**c2**) Wear charts of device performance between WFHE and rigid wristband system. Reprinted from [126], copyright (2020), with permission from Elsevier.

Flexible wearable devices tailored for the upper body are gaining prominence, serving as pivotal tools for monitoring upper limb kinematics as well as cardiac and pulmonary functions during athletic endeavors. These devices offer robust technical reinforcement for both training and competitive scenarios in sports (Figure 6).

For instance, the PowerGlove, a specialized wearable glove designed for athletes, is embedded with an ultra-thin sensor. This sensor, governed by an Arduino Nano microcontroller, captures data pertaining to the pressure exerted by the hand on a ball during specific actions such as serves or smashes [124]. Such real-time feedback facilitates nuanced analysis, enabling athletes to refine their technical maneuvers, thereby augmenting their overall performance. Moreover, while wearable devices like the Polar H10 heart belt, Polar Vantage V2, and Garmin Venu Sq smartwatches, as highlighted by G. Cosoli (2022), exhibit certain limitations, they remain invaluable in furnishing data that can guide athletes in optimizing their training regimens [125]. Ye (2020) introduced a wearable biosensor that offers real-time, non-invasive, and non-irritant monitoring of physiological states [130]. This device, interfacing with the human epidermis, leverages sweat, and interstitial fluid to gauge lactate levels during physical activity, providing insights into exercise efficacy.

Recent advancements have seen the integration of sweat sensors into upper-body garments, circumventing the need for invasive implants [22,131]. Consequently, devices such as the wearable acceleration sensor introduced by He (2022) and the WFHE, a malleable, wearable, flexible, hybrid electronic device, can be directly adhered to the skin [8,126]. These innovations ensure a secure and seamless attachment to the wrist’s epidermis, obviating the need for additional wristbands and ensuring consistent health and performance monitoring. Additionally, the sports domain has witnessed the emergence of flexible wearable thermal textiles tailored for winter sports. These accessories offer athletes a lightweight, comfortable, and thermally insulated solution, mitigating injury risks in frigid environments [132].

In summation, flexible wearable devices designed for the upper limbs are carving a significant niche in the sports arena. They not only amplify the training and competitive potential of athletes but also pave the way for novel research trajectories in sports science.

### 4.3. Flexible Wearable Devices Applied to the Lower Body

**Figure 7:**
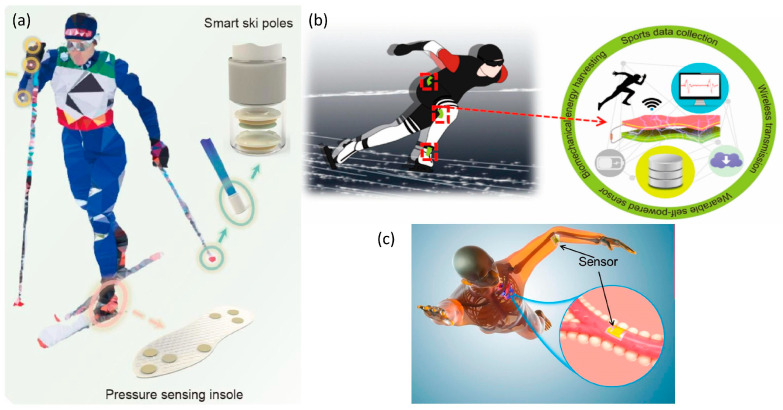
The images of flexible wearable products worn on the lower body. (**a**) Structure of the pressure-sensing insole and smart ski poles based on CF-TENG and their application in skiing sports monitoring. Reprinted from [127], copyright (2022), with permission from Springer Nature. (**b**) The application scenarios of MS-TENG. Reprinted from [128], copyright (2022), with permission from MDPI. (**c**) A potential scenario of self-powered biosensors in swimming monitoring. Reprinted from [129], copyright (2021), with permission from MDPI.

Flexible wearable devices have emerged as pivotal tools in providing sophisticated technical support for the lower body training of athletes. For instance, the innovative smart insole, conceptualized by Liu (2022), is equipped with embedded data sensors [127]. These sensors meticulously monitor the force exerted on the foot’s sole, subsequently analyzing the foot’s sole parameters. This real-time analysis aids in tracking the athlete’s physiological state offering feedback on any gait anomalies, thereby preempting potential injuries. Such instantaneous data feedback empowers athletes to dissect and refine their technical movements, culminating in enhanced athletic performance (Figure 7).

Moreover, Rajendran (2021) discerned a correlation between athletes’ psychological challenges and gait imbalances [133]. By monitoring athletes’ gait distribution, potential psychological impediments can be identified, paving the way for optimized competitive performance. The integration of these devices augments the scientific rigor and precision of athletes’ training regimens.

Further advancements in wearable technology have led to the development of sensors that seamlessly adhere to the skin. Lu (2022) developed the triboelectric nanogenerator (TENG), amalgamated with sensors strategically positioned on pivotal joints, including the hip, thigh, and ankle, specially tailored for speed skaters [128]. This avant-garde device transduces athletes’ technical maneuvers, such as body inclination angles and thigh oscillation frequencies during activities like running and skating, into electrical signals. These signals are subsequently relayed to a data hub for comprehensive analysis, facilitating the creation of bespoke training blueprints and amplifying training efficacy. In a parallel vein, Mao (2021) introduced a self-sustaining biosensor fortified with a ZnO nanowire array (ZnO NWs) [129]. This groundbreaking invention addresses the perennial challenge of energizing subaqueous wearable devices, enabling the meticulous monitoring of swimming techniques, and fostering a more scientific and energy-efficient training approach.

Presently, the research trajectory for lower-limb wearable devices predominantly orbits around kinematic indicator monitoring. A plethora of studies are dedicated to enhancing the functionalities of smart insoles, given their widespread acceptance owing to their versatility and user-friendliness. However, there remains a paucity of research delving into the physiological monitoring of the lower limbs, primarily due to the intricacies and comfort considerations associated with the wearable technology employed.

## 5. Conclusions and Future Perspectives

In the realm of sports science, flexible wearable devices have emerged as indispensable tools. The incorporation of microfluidic chips into these devices offers the potential to mitigate extraneous variables during physical activity, thereby ensuring more precise monitoring outcomes [18]. The multifaceted wearable devices developed to date, whether tailored for the head, upper limbs, or lower limbs, furnish comprehensive technical support for both athlete training and competitive endeavors.

The trajectory of flexible wearable device applications is poised for broader and more profound exploration. The integration of microfluidic chips is anticipated to augment the precision, comfort, and multifunctionality of these wearable devices. Consequently, this will pave the way for meticulous personalized guidance, benefiting not only elite athletes but also a broader demographic, underscoring the transformative potential of this technology in the sports domain.

## Figures and Tables

**Table 1:** Comparison of tear test and sweat test products.

Product Name	Biomarker Composition	Product Components	Sensitivity	Monitoring Limitations
Electrochemical Tattoo Biosensors [23]	Lactic acid in sweat.	A mediated lactate oxidase (LOx) working electrode, tetrathiafulvalene (TTF), multi-walled carbon nanotubes (CNTs), and a biocompatible chitosan layer.	The biosensor demonstrates chemoselectivity towards lactic acid with a linear response up to 20 mM.	The biosensor displays robustness against mechanical deformation typical of skin abrasion, qualifying it for extended monitoring during physical exertion.
Printed sensors [25]	Lactic acid, Na+, and NH4+ in sweat.	The sensor for lactate utilized a working electrode based on lactate oxidase, mediated by tetrathiafulvalene.	Lactate sensor utilized a diffusion-limited polyvinyl chloride membrane, and demonstrated a sensitivity of 3.28 mamM and a linear range of up to 20 mM lactate.	Control electric potential change.
Real-time colorimetric hydration sensor [26]	Hydration levels in buffers and synthetic sweat.	The colorimetric sensor utilized gold nanoparticles (AUNP) as the sensing material, which are coated with ascorbic acid to detect sodium cations in diverse hydration states.	The colorimetric sensor utilizing gold nanoparticles (AUNPs) exhibited rapid response times.	Dehydration and over-hydration
Contact lens (CL) biosensor [35]	Glucose in tears	Micro-electro-mechanical systems.	Coating with 3% PMEH showed an optimal trade-off in sensitivity and reproducibility.	Biochemical components in tear fluids.
Multifunctional contact lens [36]	Glucose in tears and intraocular pressure	Glucose sensors used in it based on the FET consist of the graphene channel and hybrid S/D	Immobilized on the graphene channel using a pyrene linker via p–p stacking.	Molecular concentrations at the already impermeable graphene surface.
Wearable tear bioelectronic platform [37]	Glucose in tears and vitamins	On-line fluidic device; an alcohol oxidase (AOx)-based electrochemical detector	Insights into the effect of the tear stimulation and collection upon the analyte concentration and of the detector geometry	High BAC measurements, due to alcohol remaining in the oral cavity.

**Table 2:** Details pertaining to flexible wearable devices.

Area of the Body	Device Name	Function
Head	Smart glasses [37]	Monitor sweat metabolites and electrolytes
	Mouthguard biosensors [120]	Real-time monitoring of salivary uric acid levels
	Smart face mask with heat flux sensor [121]	Non-invasive body temperature and breathing rate monitoring
	Smart face mask for wireless CO_2_ monitoring [122]	Monitor CO_2_ in DSV
	Lab-on-Mask [123]	Monitor cardio-respiratory variables
Upper body	PowerGlove [124]	Detect the pressure data exerted by the hand on the ball during serving or smashing, helping to analyze and improve technical movements
	Polar H10; Polar Vantage V2; Garmin Venu Sq [125]	Take a heart rate assessment, and help optimize athletes’ training programs
	WFHE [126]	Help athletes with health and performance monitoring
Lower body	Pressure sensors in shoe insole [127]	Body condition monitoring and gait analysis
	Skin-attached flexible sensor [128]	Help analyze technical movements and adjust training plans
	Self-Powered Biosensors [129]	Monitor swimming action

## Data Availability

Not applicable.
